# Insights into a viral motor: the structure of the HK97 packaging termination assembly

**DOI:** 10.1093/nar/gkad480

**Published:** 2023-06-09

**Authors:** Dorothy E D P Hawkins, Oliver W Bayfield, Herman K H Fung, Daniel N Grba, Alexis Huet, James F Conway, Alfred A Antson

**Affiliations:** York Structural Biology Laboratory, Department of Chemistry, University of York, York YO10 5DD, UK; York Structural Biology Laboratory, Department of Chemistry, University of York, York YO10 5DD, UK; Structural and Computational Biology Unit, European Molecular Biology Laboratory, 69117Heidelberg, Germany; MRC Mitochondrial Biology Unit, University of Cambridge, The Keith Peters Building, Cambridge Biomedical Campus, Hills Road, Cambridge CB2 0XY, UK; Department of Structural Biology, School of Medicine, University of Pittsburgh, Pittsburgh, PA 15260, USA; Department of Structural Biology, School of Medicine, University of Pittsburgh, Pittsburgh, PA 15260, USA; York Structural Biology Laboratory, Department of Chemistry, University of York, York YO10 5DD, UK

## Abstract

Double-stranded DNA viruses utilise machinery, made of terminase proteins, to package viral DNA into the capsid. For *cos* bacteriophage, a defined signal, recognised by small terminase, flanks each genome unit. Here we present the first structural data for a *cos* virus DNA packaging motor, assembled from the bacteriophage HK97 terminase proteins, procapsids encompassing the portal protein, and DNA containing a *cos* site. The cryo-EM structure is consistent with the packaging termination state adopted after DNA cleavage, with DNA density within the large terminase assembly ending abruptly at the portal protein entrance. Retention of the large terminase complex after cleavage of the short DNA substrate suggests that motor dissociation from the capsid requires headful pressure, in common with *pac* viruses. Interestingly, the clip domain of the 12-subunit portal protein does not adhere to C_12_ symmetry, indicating asymmetry induced by binding of the large terminase/DNA. The motor assembly is also highly asymmetric, showing a ring of 5 large terminase monomers, tilted against the portal. Variable degrees of extension between N- and C-terminal domains of individual subunits suggest a mechanism of DNA translocation driven by inter-domain contraction and relaxation.

## Graphical Abstract

**Graphical Abstract:**
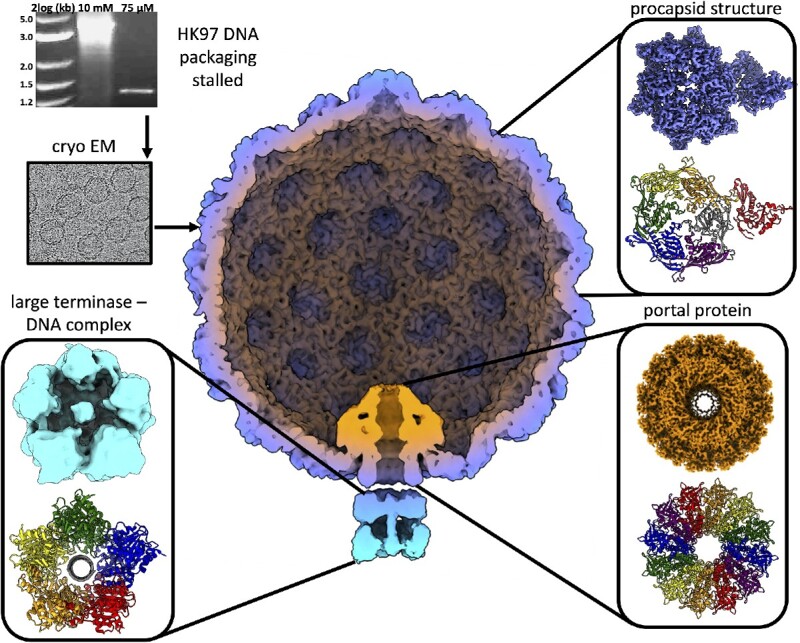

## INTRODUCTION

Encapsulation of the genome represents a key step in viral assembly and life cycle. For double stranded (ds) DNA bacteriophage, the genome is packaged into a preformed protein shell, or capsid, through the dodecameric portal protein, which acts as a door (1)⁠. Packaging often produces expansion of the capsid from an immature form, known as a prohead, to a larger, fully mature state.

Translocation of DNA against mounting internal pressure requires ATPase activity, provided by a ring of five large terminase subunits (2–6). Each monomer constitutes an N-terminal ATPase (NTD) (28) and C-terminal endonuclease domain (CTD) adjoined by a linker. This large terminase motor is thought to reach forces of up to 100 pN (7–9) and thus represents the most powerful biological machine studied. Large terminase functions akin to other ring-shaped, oligomeric translocases, which also utilise ATP hydrolysis cycles to power translocation through the central pore (10). Bacteriophages also employ small terminase proteins for recognition of the viral genome (11–14)⁠. Small terminases have also been shown to stimulate large terminase packaging activity (15–17).

Although mechanisms of packaging are broadly conserved among the dsDNA phages, further differentiations can be highlighted based on processing of the viral DNA at the initiation and termination of packaging. *Cos* viruses, such as λ, P2, and HK97—the subject of this paper—package exact genome-length DNA units (18,19), separated by consecutive *cos* (cohesive) sites in a genome concatemer (20). For *pac* viruses, including P22, P1, SPP1 and T4, packaging is initiated from a *pac* (packaging) site within the genome (21–23) and terminates in response to ‘headful’ pressure within the prohead, leading to encapsulated genomes varying in length from ∼103 to 110% (24). Finally, Φ29-like phages produce unit-length DNA genome copies, where each 5′ end is covalently bound to viral protein gp3, which facilitates packaging (25)⁠.

Portal proteins display highly divergent sequences and molecular weights, but *in situ* within the viral capsid, consistently present as dodecameric rings (26–28). The monomeric portal protein structures share conserved protein folds and domain arrangement comprising the clip, stem, wing and crown domains. Positioned at the base of the portal protein, the clip domain interacts with the large terminase motor during assembly, and later the adaptor proteins for tail attachment (26). The stem contains the ‘tunnel’ α-helix that lines the internal channel with several negatively charged residues (29). The most structurally divergent domain, the wing, coordinates contact with the capsid proteins (26–28). Lastly the crown, exposed at the inner surface of the capsid, interacts with packaged DNA (30).

The portal protein displays remarkable plasticity, proposed to facilitate symmetry-mismatching interactions with the capsid and terminase. This flexibility also permits propagation of pressure changes within the capsid to the terminase (31), in order to induce packaging termination. Indeed, the DNA in mature phage P22, is spooled tightly around the portal in an arrangement which appears to be incompatible with the procapsid portal form (31). Meanwhile several discrete mutations within the P22 and *λ* portal cores produce over-packing phenotypes (32,33), indicating unsuccessful termination. Conformational changes within the portal protein during DNA packaging appear to be a conserved feature among dsDNA viruses, with marked variability between procapsid and mature head portal structures for T7, Φ29, p22 and P23-45 (30,31,34–38).

Much mechanistic understanding of the large terminase motor has been drawn from single molecule optical tweezer studies on the phage Φ29. The Φ29 large terminase mechano-chemical cycle has been shown to alternate between two phases. During the dwell phase ATP binds cooperatively to each subunit around the ATPase ring, and the DNA substrate remains stationary. During the burst phase, DNA translocation into the capsid occurs in four subsequent 2.5 base pair steps, corresponding to four ATP hydrolysis events (39). A cryo-EM reconstruction of the intact Φ29 packaging motor, comprising the capsid, pRNA, large terminase and DNA, revealed the five ATPase domains, in a ‘cracked’ helical conformation, stabilised by ATP*γ*S (40).This conformation contrasts planar structures seen for the apo Φ29 ATPase (41,42) and the ADP-bound form of closely related phage ascc-φ28 large terminase (43). The transition between the cracked helical and planar states likely represents the burst phase, which has inspired a unique DNA translocation model (43). However, other terminase-packaging systems may well adopt mechanisms different to Φ29, which unusually utilises pRNA and does not require small terminase protein (42,44).

Whilst a wealth of structural data comprising individual packaging proteins has emerged for dsDNA viruses, the field is only starting to move towards analysing complete packaging systems. For bacteriophage HK97, the focus of present study, structural information is so far available only for individual components including large and small terminase proteins (45) and prohead II (the immature prohead state at packaging initiation) (46). The X-ray structure of monomeric large terminase revealed the classical ATPase and nuclease domains adjoined by a short linker (45,47). In common with most other phage, the small terminase contains 9 subunits arranged in a circular structure exposing N-terminal HTH motifs around the outside of the oligomerisation core of the molecule, where they form a positively charged rim (36,38). ⁠

The cryo-EM structure of the active HK97 packaging machinery presented here, in combination with previous work establishing a functional DNA packaging assay (45), begins to shed light on a number of unanswered questions for *cos* phage: (i) how do these viruses overcome the symmetry mismatch between the 12-subunit portal and pentameric large terminase, (ii) does small terminase remain engaged with the motor throughout packaging and (iii) what is the mechanism of coordinated ATP hydrolysis around the large terminase ring and how are the chemical events of ATP hydrolysis coupled to mechanical translocation of DNA.

## MATERIALS AND METHODS

### Packaging assays

Individual packaging protein components of the packaging assays were expressed and purified as described (45). The DNA substrate used represented a linearised pUC18 plasmid with an engineered *cos* site from –312 to +472 of the cleavage sites. Limiting the packaging time from 30 to 2 min significantly improved large terminase retention, and the addition of DNAase1 to the final sample improved the background signal.

### Data collection

R 3.5/1 Quantifoil, with 2 nm Ultrathin Carbon, 200 mesh copper grids were glow discharged for 60 s at 15 mA in a PELCO easiGlow™. A 3 μl sample was prepped immediately prior to vitrifying using the Vitribot IV, with blot force –5 and blot time 2 s (54). This grid was subject to a 72-h data collection at the UK National eBIC (Electron Bio-Imaging Centre) at the Diamond Light Source (Harwell Science and Innovation Campus) on an FEI Titan Krios instrument using a K3 detector. The data collection parameters are summarised in Supplementary Table S1.

### Reconstruction of the prohead/portal/motor complex (Fig S1)

Data were processed in RELION 3.1 (48). Motion correction was performed using MotionCor2 (49,50) and corrected micrographs were then subject to CTF estimation using CTFFIND 4 (51). Optimised picking parameters were achieved using Topaz, as an external RELION job (52). A total of 352 600 particles were picked and subject to 2D classification. The best classes were selected, and duplicates removed, leaving 82 279 particles for icosahedral refinement. This map was subject to post processing and CTF refinement before a second round of refinement (53) (Supplementary Figure S1A). The resolution of the final post processed reconstruction is 3.06 Å (FSC = 0.143).

During icosahedral refinement signal from asymmetric features of the virus is averaged out evenly over the map. Thus, in order to model the portal and motor, the icosahedral 3D reconstruction of the combined data sets was first subject to RELION symmetry expansion (53)(in symmetry point group I3), which generates a 60-fold increased set of particles. Knowing the location of the vertices, we could determine new extraction coordinates (54) for re-extraction of each vertex whilst binning to 5.2 Å/pixel. This produced 60 subparticles per capsid particle. Subparticles were subject to 3D classification without alignment to separate subparticles containing portal and motor signal, from pentameric capsomers. Initially a cylindrical mask, created using relion_helix_toolbox (55) with a soft edge of 2 pixels and extension of 2 pixels, was used to mask out contributing signal from the capsid, centred on the expected position for the portal and motor. One clear portal-containing class emerged, which was used as a template for a tighter mask applied to the original subparticle set for further classification.

To reconstruct the portal protein structure, particles from the portal-motor class were selected and subjected to 3D classification without performing image alignment. The classes displaying the most defined secondary structure were selected and re-extracted into a smaller box encompassing only the portal (1.34 Å/pixel). These 57279 particles were refined using C12 symmetry. The 3D map was then subject to postprocessing reaching 2.98 Å resolution (FSC = 0.143) and local resolution estimation to calculate resolution within different local sections of the map (Supplementary Figure S1B).

The motor signal was isolated by re-extraction of particles at the portal vertex into a smaller box size encompassing the motor density only. These were subject to 3D classification without performing image alignment, and classes displaying clear individual subunits and domains were subject to a second classification into a single class. This was used as a template for a mask used for particle subtraction. This class contained just 17584 particles. Subtracted particles were subsequently subject to refinement with local angular searches (1.8 °) followed by post-processing reaching a final resolution of 8.8 Å (FSC = 1.43) (Supplementary Figure S1C).

The run_data.star file from the motor refinement was used as a template for re-extraction to include portal density. The same protocol of particle subtraction was employed, using a mask to encompass the whole portal/motor complex. Subtracted particles were then refined with limited angular searches of 1.8°. After postprocessing the resolution of the portal/large terminase complex reached 7.4 Å (Supplementary Figure S1C). The portal/motor complex was then reextracted to include the entire prohead and subject to symmetry expansion in C_12_. Particles were subject to 3D classification without alignment and the best five classes selected. Duplicates were removed leaving 8354 particles for 3D refinement (Supplementary Figure S1C). The resolution of the complete packaging complex was 8.3 Å (Supplementary Figure S1C).

### Model building

Portal and prohead atomic models were built in Coot 0.8.9.3 (56) and refined using Phenix 1.19 Cryo EM Real Space Refinement (57). PDB model 3E8K (46) was used as a starting model for the asymmetric prohead unit. Residues 130–382 were refined for each of the 7 chains. The first 103 residues are cleaved off in the transition from Prohead I, and density for residues 104–129 was not sufficiently defined for model building suggesting flexibility (58). For the portal protein, a starting model for a single chain was established using an AlphaFold prediction (59) using uniprot reference P49859. This was symmetrised 12-fold, and residues 32–398 refined into the C_12_ map. Model validation parameters are summarised in Supplementary Supplementary Table S2.

## RESULTS

### Stalling and stabilising the DNA packaging motor

**Figure 1.**
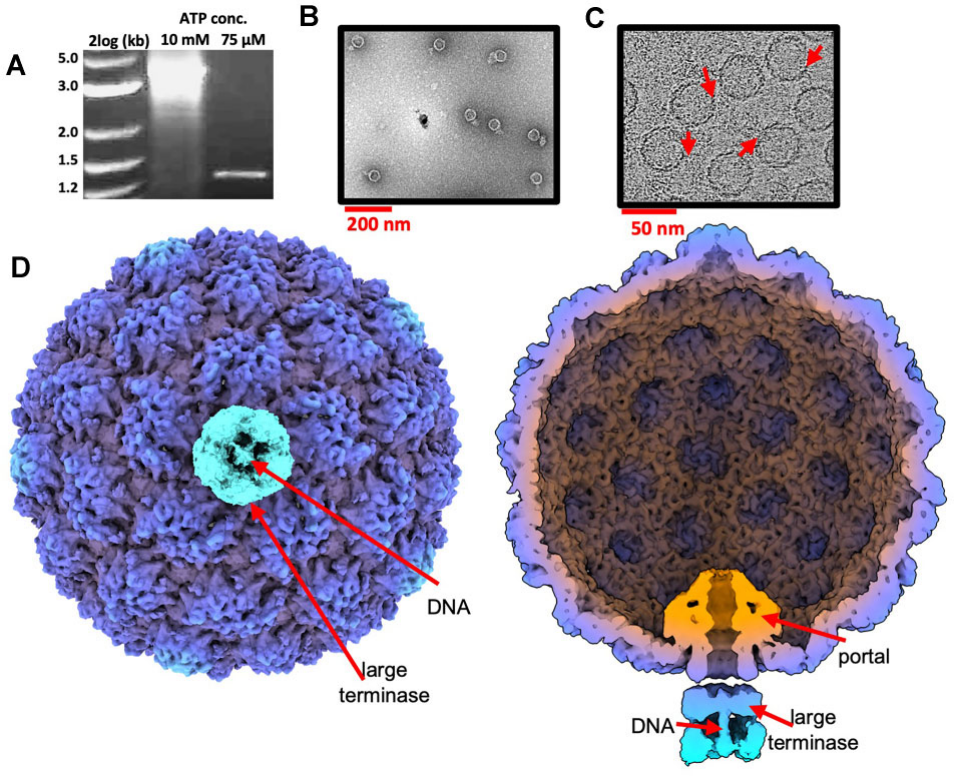
Stalled HK97 packaging complexes. (**A**) DNAse protection assay for the HK97 packaging motor using a cos containing substrate at variable ATP concentrations. (**B**) Sample visualised by negative stain electron microscopy. (**C**) Sample visualised by cryo-EM. Red arrows highlight DNA packaging centres. (**D**) External and cross-sectional views of the asymmetric reconstruction.

*In vitro*, the HK97 DNA packaging motor has been shown to stall at a low ATP concentration if an internal *cos* site is incorporated into the DNA substrate (45). This stalling behaviour was deemed desirable for structural characterisation, potentially allowing the motor to be ‘fixed’ in a single conformation, preventing unsynchronised packaging and motor detachment. Thus, DNA protection assays were utilised to examine optimal stalling conditions: small terminase was critical, and the ideal ATP concentration was pinpointed to 75 μM (Figure 1A). The optimised sample was then flooded with 5 mM ATP$\gamma$S and visualised by negative stain electron microscopy (Figure 1B) to check for associated packaging motors. The 3D structure of stalled motor was derived by cryo-electron microscopy (Figure 1C) and single-particle analysis. An asymmetric reconstruction comprising prohead II and the portal/large terminase complex, was derived at 8.3 Å. Despite limited resolution, the reconstruction contained well defined density at the unique portal vertex, likely corresponding to a large terminase oligomer. Local refinement of each protein constituent is described subsequently.

### Structure of HK97 prohead II

**Figure 2.**
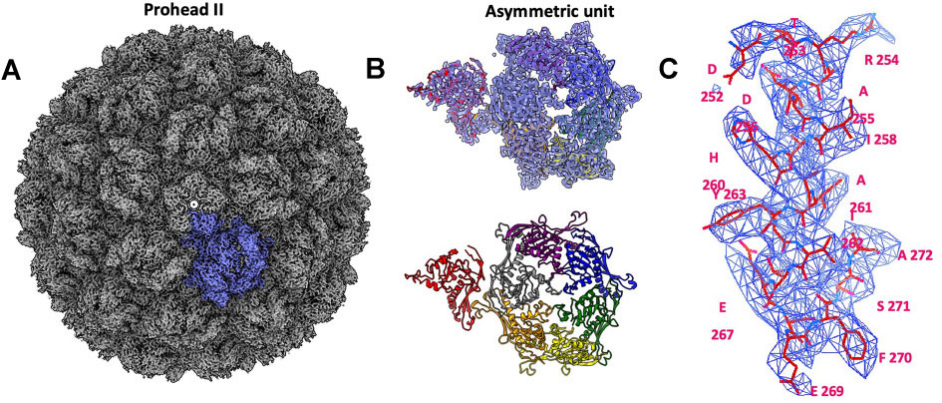
Icosahedral reconstruction of the HK97 prohead II at 3.06 Å resolution. (**A**) EM density viewed along the five-fold axis. (**B**) Ribbon diagram of the asymmetric capsomere unit shown alone (bottom) and fit into the corresponding density map (top). (**C**) Representative EM map shown for a prohead segment fitted with corresponding atomic model.

The cryo-EM structure of the prohead was determined at 3.06 Å using icosahedral averaging (Figure 2). EM density (Figure 2C) allowed for refinement of the previously determined 3.6 Å crystal structure (46). The prohead measures 550 Å in diameter and shows dislocated or ‘skewed’ trimers within the hexamers (Figure 2 A). This is consistent with structures of prohead II (46), depicting a stage of capsid maturation prior to expansion and after cleavage of the scaffolding domains from the major capsid protein. This indicates that proheads remain unexpanded during packaging and stalling. Importantly, unlike the earlier reported crystal structure (46) the present structure was derived for proheads containing the packaging machinery, representing a biologically relevant form.

### Structure of HK97 portal protein

**Figure 3.**
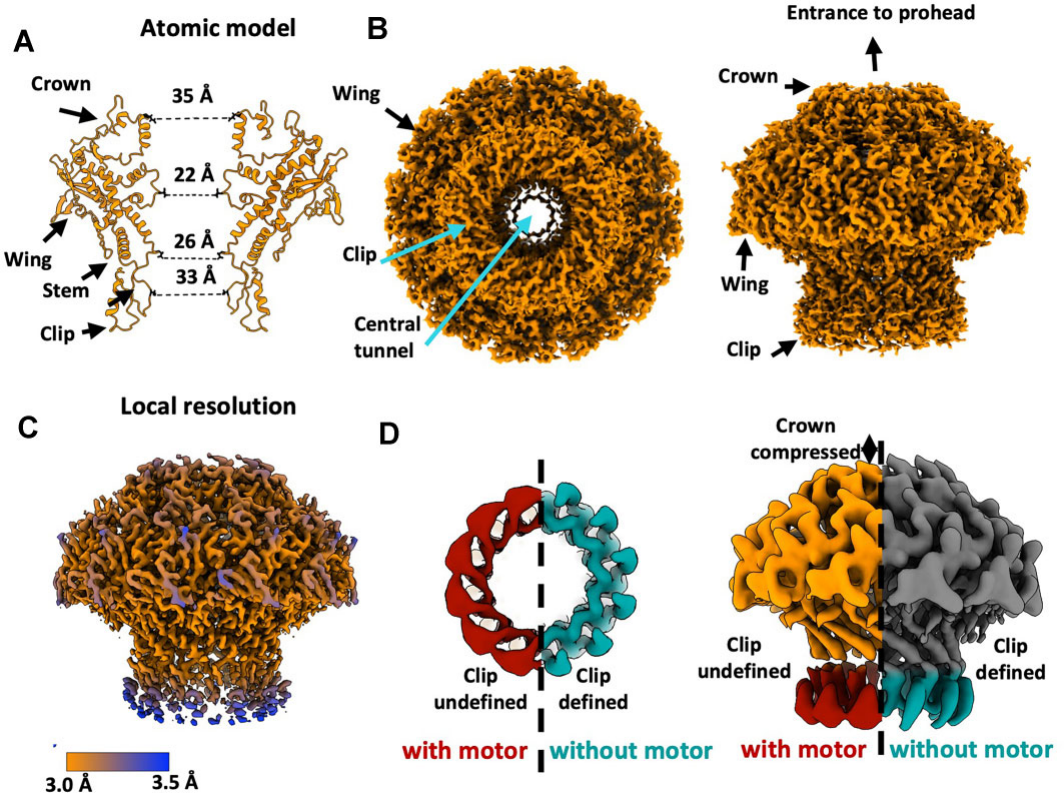
Structure of the portal protein. (**A–C**) C_12_ reconstruction of the in-situ portal protein at 2.98 Å resolution. (A) Ribbon diagram with only two opposing subunits. (B, C) Portal density. (**D**) Comparison of portal protein maps in the presence and absence of the motor, viewed from the clip (left) and perpendicular to the portal axis (right).

The portal protein reconstruction shows clear 12-fold symmetry with an overall ‘mushroom’ shaped architecture (Figure 3). An atomic model was built into the 2.98 Å resolution map. The clip, stem, and crown domains line an extended DNA channel, with the wing domain spanning outwards (Figure 3B). The diameter of the central channel varies from ∼35 Å at the crown domain, to ∼33 Å in the clip domain, and ∼22 Å in the tightest part of the stem (Figure 3A). This is sufficiently wide for translocating B-form DNA (60). The local resolution of the portal protein (Figure 3C) shows the wing domain particularly well resolved, with lower resolution for the crown and especially the clip domain. A reconstruction of the portal protein from a separate sample of HK97 prohead II featuring the *in situ* portal protein, but without terminase (unpublished data), presents an informative comparison (Figure 3D). While the global resolution of this ‘empty prohead’ portal is lower, at 4.1 Å, low-pass filtering each map to the same threshold indicates a distinct improvement in the definition of the clip domain, where density corresponding to α-helices can be clearly distinguished. Further differences appear also in the crown domain, which is extended in terminase absence.

### Reconstruction of the packaging motor comprising the portal-large terminase complex

In spite of attempts to fix the large terminase motor in a single state—both by introducing a *cos* site in the DNA substrate and providing a high concentration of ATP$\gamma$S— processing of the data indicates that the motor is highly flexible and heterogeneous. Icosahedral symmetry expansion of the capsid followed by focussed 3D classification produced two broad portal/large terminase classes. Both showed strong signal at the large terminase locus but little definition, indicative of flexibility (Supplementary Figure S1B, C). Indeed, further 3D classification revealed a whole spectrum of conformation, only several of which displayed defined subunits (Supplementary Figure S1C). Subsequently, the terminase oligomer reconstruction represents just 17 584 particles out of a total of approximately 82 279 portal/large terminase particles.

**Figure 4.**
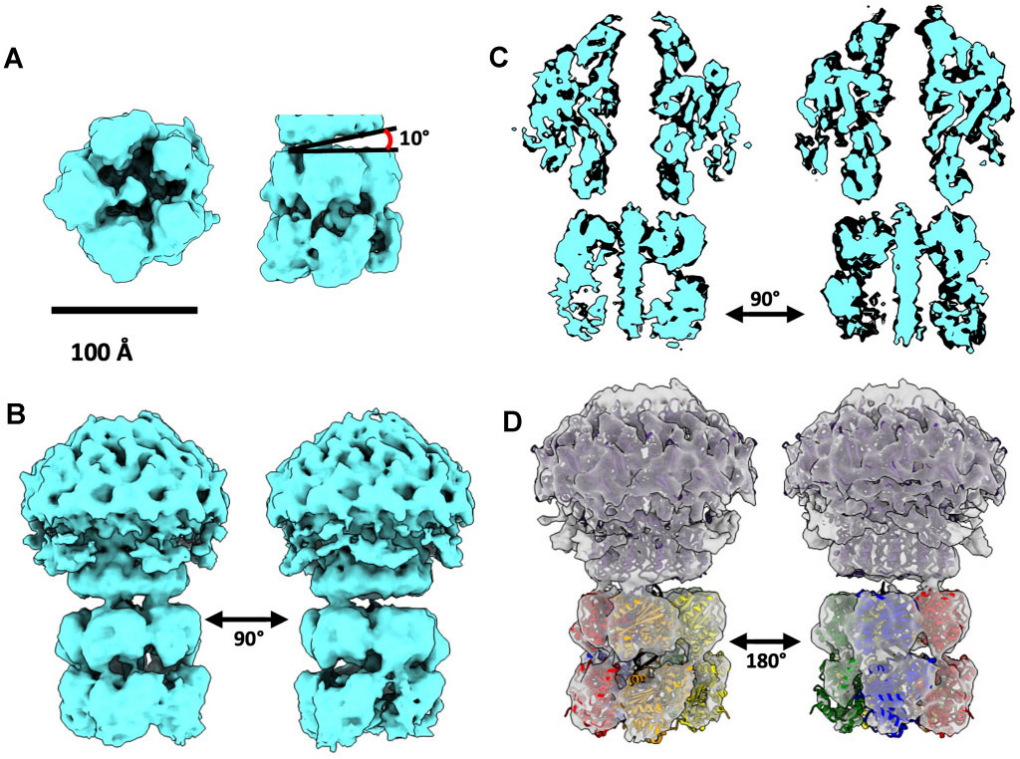
Asymmetric reconstruction of the HK97 DNA packaging motor. (**A**) Pentameric motor surrounding DNA substrate. The terminase ring is tilted 10° relative to the portal. (**B**) The portal-terminase motor complex. (**C**) Cross sectional views of the portal-motor complex. (**D**) Result of the rigid body docking of terminase domains, derived from the crystal structure of the HK97 large terminase, into the EM density with the fitted models shown as ribbon diagrams coloured by subunit.

The asymmetric reconstruction, estimates at 8.8 Å resolution (Figure 4A), allows the five large terminase subunits to be clearly observed, which are arranged in a pentameric ring encircling the dsDNA substrate. This stoichiometry for large terminase is in keeping with previous fluorescent photobleaching experiments (45) and the motor assemblies of other dsDNA bacteriophages (2,4–6,40). When the same particles were re-extracted to encompass both the large terminase and the portal protein in complex, the resolution improved to 7.4 Å (Figure 4B), consistent with a more structurally rigid conformation. Clearly resolved rod-like density for the DNA within the lumen of the large terminase appears to make contact with both the N- and C-terminus domains of large terminase monomers in both reconstructions (Figure 4).

The large terminase reconstruction is in good agreement with the crystal structure of the monomer (45) with densities for each ATPase and nuclease domain well resolved (Figure 4A–C). Flexibility within the complex is likely confined to inter-domain and inter-subunit movement, as opposed to domain rearrangement, consistent with the globular nature of the two domains and the ability to crystallise single-domain constructs of related large terminases more readily than full-length constructs (61–67). Thus, individual domains were subject to rigid-body fitting into the density (57) (Figure 4D). The more compact nuclease domains were fitted into the ring directly below the portal, with the N-terminal ATPase domains positioned more flexibly beneath, with ∼25 bp DNA spanning the central tunnel of the terminase assembly.

## DISCUSSION

### Motor assembly induces asymmetry in the portal protein

The density corresponding to the portal clip domain within the packaging complex, compared to the portal clip domain in the empty prohead, is poorly resolved. This suggests distortion away from C_12_ symmetry during packaging. We hypothesise that the plasticity of the clip domain compensates for the symmetry mismatch at the portal-large terminase interface and facilitates interactions with the large terminase. Meanwhile, the reduced height of the crown domain in the portal/large terminase complex echoes structural changes in related portal proteins, where the mounting pressure from DNA within the capsid is thought to induce portal compression (34,37). Flexibility within each of these domains has been indicated in studies of other viruses during DNA packaging (37,68).

### Small terminase likely dissociates after inducing large terminase endonuclease activity

**Figure 5.**
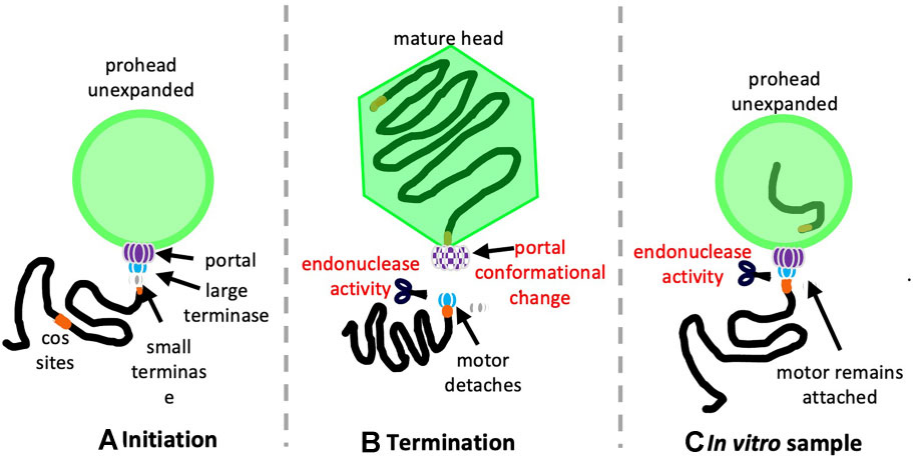
Packaging termination *in vivo* and *in vitro*. (**A**) Initiation complex of HK97 DNA packaging *in vivo*. (**B**) Termination complex of HK97 packaging *in vivo*. (**C**) HK97 stalled packaging complex present *in vitro*, after cleavage of DNA with the large terminase assembly remaining attached, releasing the cleaved DNA into the prohead interior.

Small terminase is essential for the stalling of the HK97 large terminase motor, and thus present in the sample, but no obvious density corresponding to a small terminase is apparent within the density maps. The nonameric ring of small terminase has a molecular weight of 160 kDa, which should be discernible even if positioned flexibly. This indicates a transient role for small terminase in the complex formation. We hypothesise that the small terminase remains bound to this downstream *cos* site throughout packaging, acting as a roadblock for incoming large terminase, and instigating the conformational change required to switch the motor from displaying ATPase activity to endonuclease activity. In turn, small terminase may dissociate from the DNA (Figure 5C). The documented stimulatory effect of the small terminase (45), in this model, would then be attributed solely to recruitment of large terminase to the viral DNA, thus enhancing the number of packaging initiation events.

### DNA cleavage at the large terminase-portal interface mimics termination

One striking feature of the portal-large terminase map is the apparent lack of DNA density within the portal channel (Figure 4C). DNA extends through the lumen formed by the large terminase oligomer and stops at the interface between the large terminase and portal. The length of this DNA is consistent with approximately 25 bp of B-form DNA. Packaging assays consistently show protected DNA within proheads of ∼1.3 kbp, suggesting that the motor has packaged from the 5′ end of the DNA substrate and paused at the *cos* site (Figure 1A).

This conformation mimics the packaging termination state and indicates that on reaching a *cos* site after packaging one complete genome unit, large terminase endonuclease activity was activated. The nuclease domain is expected to employ the two-metal catalysis mechanism, as it adopts the classical RNase H family fold, with closest similarity to the RuvC endonucleases (62,63,65,69). Docking five copies of the HK97 large terminase crystal structure into the oligomeric density (Figure 4D) shows that each subunit is oriented with the nuclease active site exposed towards the cleaved DNA end. These structural observations are consistent with the observed cut in the DNA and the termination state of the motor.

*In vivo*, DNA cleavage is expected to trigger dissociation of the motor allowing for tail attachment (70), Figure 5B. In our sample however, large terminase remains bound to the portal (Figure 5C). This suggests that packaging termination may occur *via* two distinct steps, controlled by different signals: (i) DNA cleavage at a *cos* site is mediated by small terminase large terminase interactions; (ii) release of the large terminase occurs in response to headful pressure signalling, likely relayed through the portal. In the structure of prohead II, presented here, the capsid remains relatively empty, and unexpanded, as the ∼1.3 kb length of DNA inside represents just ∼3% of 39.7 kb genome. In the absence of the headful signalling the large terminase hence does not detach, but cleavage at the *cos* site can still occur (Figure 5).

### Translocation is mediated by interdomain extension-contraction

An asymmetric interaction between the large terminase and the portal clip domain is apparent in the portal-large terminase reconstruction (Figure 4). Clear contact with the portal protein appears only between one large terminase subunit, with a second adjacent subunit showing limited interactions. The entire large terminase assembly is also tilted 10° to the portal tunnel axis (Figure 4A). Asymmetric alignment was similarly observed for the Φ29 motor, where the large terminase channel is tilted at 12.5° to the portal axis (40). However, in contrast, the Φ29 portal-terminase interaction is mediated by a pRNA, which is absent in the HK97 system, while the terminase itself lacks nuclease activity (71).

**Figure 6.**
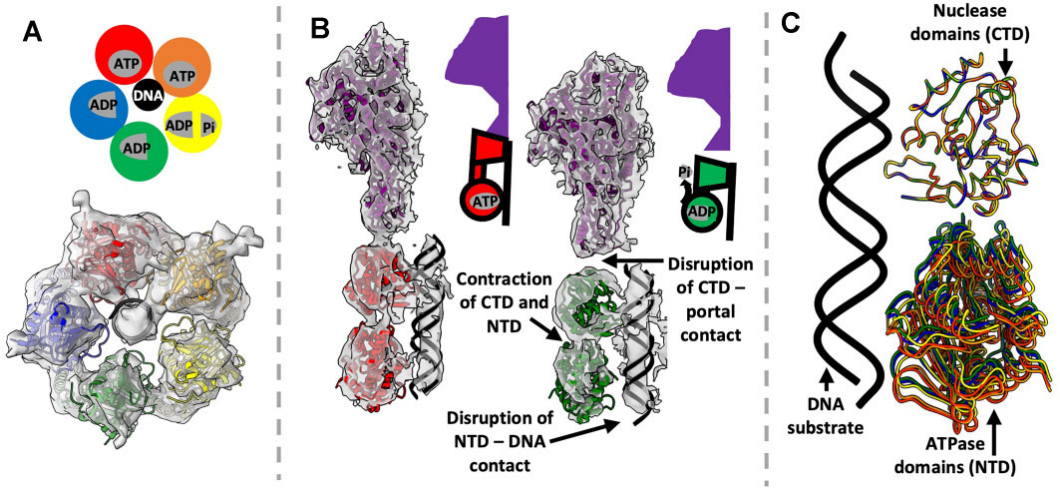
Structural comparison of HK97 large terminase monomers of the motor. (**A**) Proposed nucleoside binding pattern. (**B**) Comparison of proposed ATP and ADP bound subunits. (**C**) Overlay of large terminase monomers after aligning the nuclease domain.

In the docked crystal structure of the HK97 large terminase (Figure 4D), the two subunits which contact the portal (depicted in red and orange), are separated axially by a further ∼4.5 Å (or 1.5 bp of DNA), relative to the subunits shown in green and blue (72). Both domains of the extended subunits contact DNA (Figure 6B), whilst the contracted subunits contact DNA via the nuclease domain only (Figure 6B). Experimental data on multiple related large terminase has shown that ATP bound subunits show a high DNA binding affinity (7,73–75); and so, by analogy the extended HK97 subunits may be interpreted as ATP$\gamma$S bound whilst contracted subunits are more likely ADP bound. The presence of ADP in the sample would require slow hydrolysis of ATP$\gamma$S, as observed in DNA translocases (76). Notably, the contracted subunits display broken contact with the clip domain of the portal protein, which could potentially facilitate a cyclically changing pattern of terminase-portal interactions throughout packaging. The final large terminase subunit, shown in yellow, displays a less dramatic extension of ∼2 Å, which could correspond to mixed occupancy of the nucleotide binding site (Figure 6A) (76). An overlay of each docked large terminase ATPase domain after aligning docked nuclease domains, depicts the interdomain shifts around the pentameric ring (Figure 6C). As such, contraction and relaxation of subunits is likely involved in DNA translocation – with ATP hydrolysis coupled to subunit contraction which pushes the DNA substrate into the prohead.

**Figure 7.**
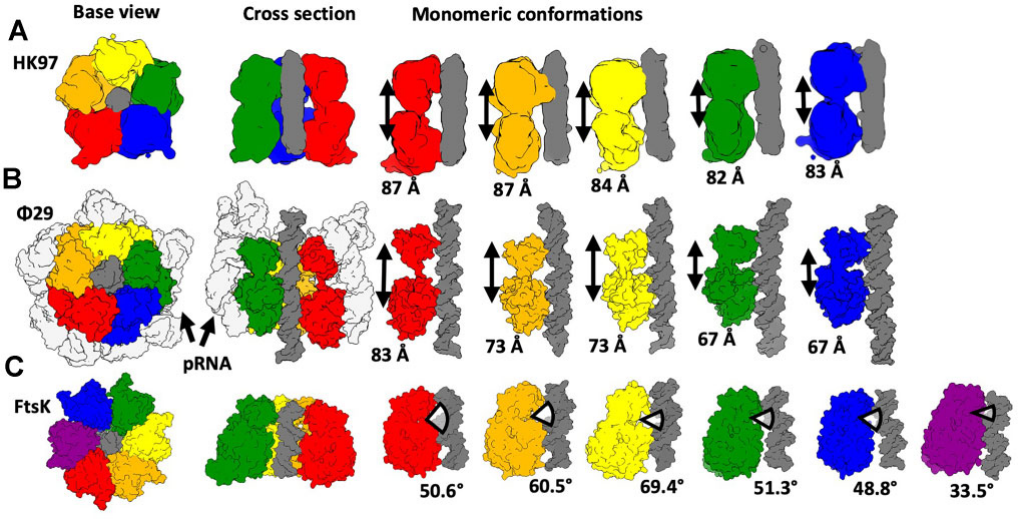
Comparison of domain adjustments in HK97, Φ29 and Ftsk DNA translocation motors. Subunits are coloured differently and shown as molecular surfaces. DNA (Φ29 and Ftsk) or DNA density (HK97) is in dark grey. For each motor (**A** – HK97, **B** – Φ29 and **C** – Ftsk) two orthogonal views of the assembly are on the left, and individual subunits along with DNA are shown on the right.

Contraction of large terminase subunits from an extended ATP bound state to a contracted ADP bound state has also been proposed as translocation mechanisms of the bacteriophages T4 and Φ29 (4,40). For T4, ATP hydrolysis is proposed to occur sequentially around the ring, so that only a single subunit is ever present in the ‘tense' contracted state (77). This does not entirely agree with the HK97 structure where subunits display a range of conformations (Figure 7A). Meanwhile, the five bacteriophage Φ29 ATPase domains are thought to make sequential shifts toward the vestigial nuclease domains (which remain in a planar ring throughout) (Figure 7B). This completes a cracked helix to planar transition, in four ATP hydrolysis steps (40). Transposition of this mechanism onto our proposed HK97 model is also problematic since transition through full ADP occupancy could destabilise the terminase-portal contact.

An alternative comparison can be made with the DNA translocation mechanism proposed for the *Escherichia coli* translocase FtsK (76). In this mechanism, a molecular machine comprises six subunits, with each adopting a unique conformation with variable angles of separation between the α and β domains (as opposed to extension), Figure 7C. Three subunits bound to ATP$\gamma$S, and a fourth with mixed ADP/ATP/APO occupancy, display DNA binding residues arranged in a spiral. The remaining two subunits are ADP-bound and do not engage with DNA (76). The proposed translocation mechanism for FtsK never passes through a fully ADP bound state, with the rolling change in conformations around the ring shifting sequentially, akin to a spiral escalator (76). This mechanism is more consistent with the HK97 structure where subunits display a range of conformations and DNA is also engaged with the subunits in extended conformation, assigned as ATP$\gamma$S bound. A comparison between the individual monomers of HK97, Φ29 and FtsK ATPases (Figure 7) shows how each system depends on a varied extension of individual subunits mediated by interdomain movements. The comparison reveals a significantly reduced range of contraction in the HK97 motor compared to Φ29. This again suggests that the HK97 motor may work akin the FtsK motor, with simultaneous conformational changes in all five terminase subunits, propagating around the ring, so that each subunit in turn adopts every conformation.

## CONCLUSIONS

Here, we present a structure of the complete packaging machinery of HK97 determined by cryo-EM. The high-resolution structure of the prohead shows that the capsid adopts the immature, unexpanded state (prohead II), present at the initiation of DNA packaging. However, the well-defined DNA density within the large terminase pentamer ends abruptly at the portal protein interface, characteristic of DNA cleavage seen at packaging termination. This suggests that packaging termination requires two distinct signals, and while the *cos* sequence present in the DNA substrate is sufficient to induce DNA cleavage by the large terminase, headful pressure is required for motor dissociation. The cryo-EM structure of the portal protein, derived at 2.98 Å resolution, shows that the clip domain alone deviates from the C_12_ symmetry, facilitating interactions with the five subunits of the large terminase. Docking of the large terminase subunits into the motor map suggests that the observed extension and contraction between the two domains of each subunit is involved in ATP-driven translocation of DNA into prohead, with both the N- and C-terminal subunits making contacts with DNA (55). This translocation model has commonalities with mechanisms proposed for the Φ29 and T4 packaging motors (40,77), as well as the DNA translocase Ftsk (76), each of which is proposed to utilise inter-subunit contraction to move DNA through the central pore of an oligomeric ring. The variable domain arrangement and the extent of observed contraction/extension of each subunit of FtsK coupled with the rolling ATP–ADP exchange, fits particularly well with the observed asymmetric nature of the HK97 large terminase pentamer, and the limited interaction with the portal protein.

## Supplementary Material

gkad480_Supplemental_File

## Data Availability

Atomic coordinates for the prohead and portal protein and maps have been deposited with the Protein Data Bank and Electron Microscopy Data Bank under accession numbers 8CFA/EMD-16624 and 8CEZ/EMD-16614 respectively. Maps for the isolated large terminase complex (Figure 4A) the portal/large terminase complex (Figure 4B,C,D), and the whole packaging complex (Figure 1D) have been deposited to the Electron Microscopy Data Bank under accession numbers EMD-16654, EMD-16653 and EMD-16649, respectively.
