# Supplementary material for: Insights into a viral motor: the structure of the HK97 packaging termination assembly
Source: Nucleic Acids Res. 2023 Jun 9;51(13):7025–35. doi: 10.1093/nar/gkad480 (PMC10359639; doi:10.1093/nar/gkad480)
Supplement: gkad480_Supplemental_File [file gkad480_supplemental_file.docx]

**SUPPLIMENTARY INFORMATION**

***Table S1: Cryo-EM data collection parameters for stalled HK97 packaging complexes***

| Location | eBIC |
| --- | --- |
| Grid type | R 3.5/1 with Ultrathin Carbon |
| Microscope | Krios 2 |
| Detector (Mode) | K3 (counting super-resolution) |
| Accelerating Voltage (kV) | 300 |
| Spherical aberration / mm | 2.7 |
| Calibrated pixel size (Å) | 1.34 |
| Nominal Mag. | 64,000x |
| Total dose (eÅ^-2^) | 40 |
| Nominal defocus range (µm) | -0.5 to -2.5 |

**
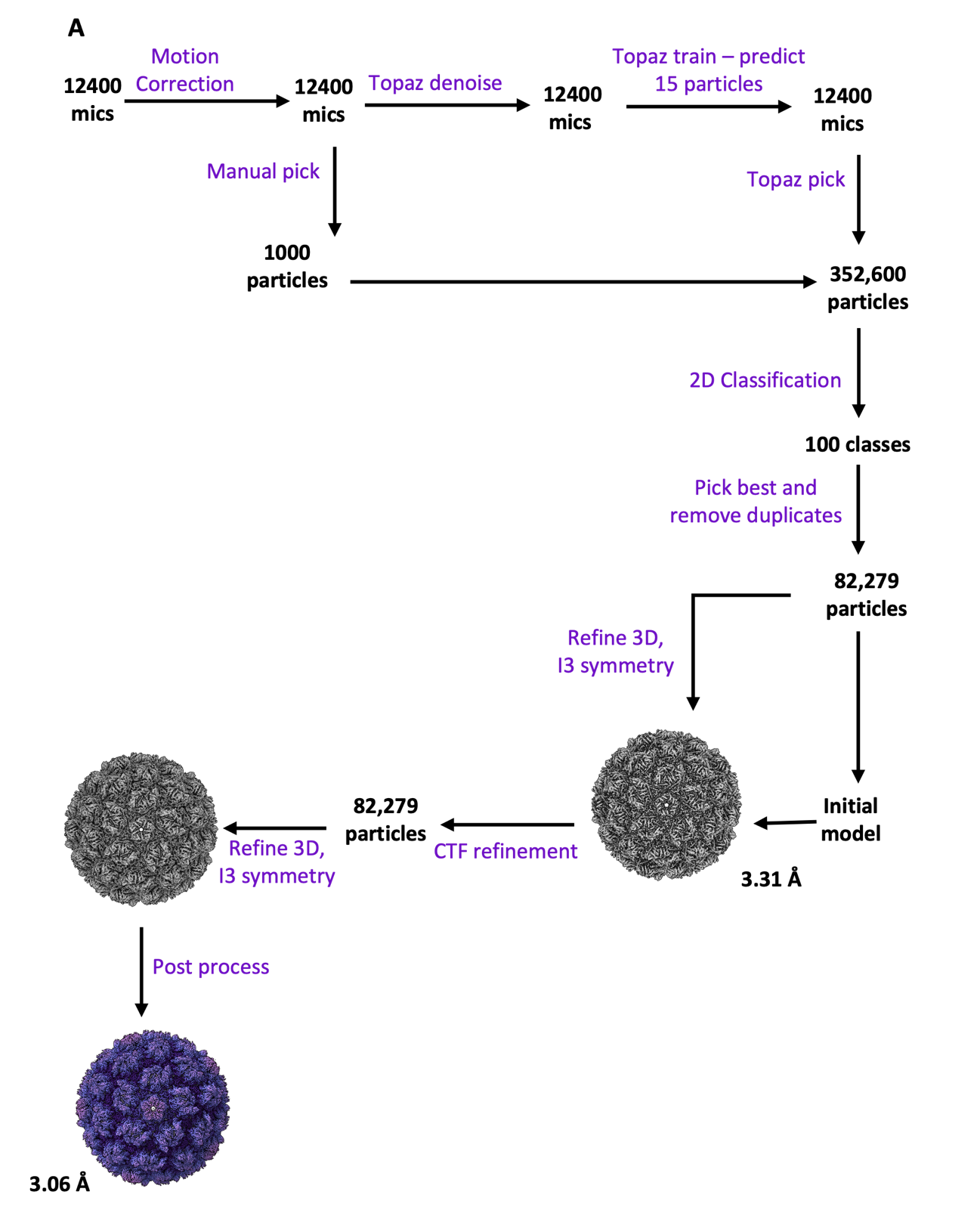
**

***Figure S1: Data processing schemes*** *for* ***A*** *- the HK97 prohead,* ***B*** *- the HK97 portal protein and* ***C*** *- the HK97 large terminase assembly, portal/terminase complex and complete packaging complex comprising prohead II, portal protein and large terminase.*

**
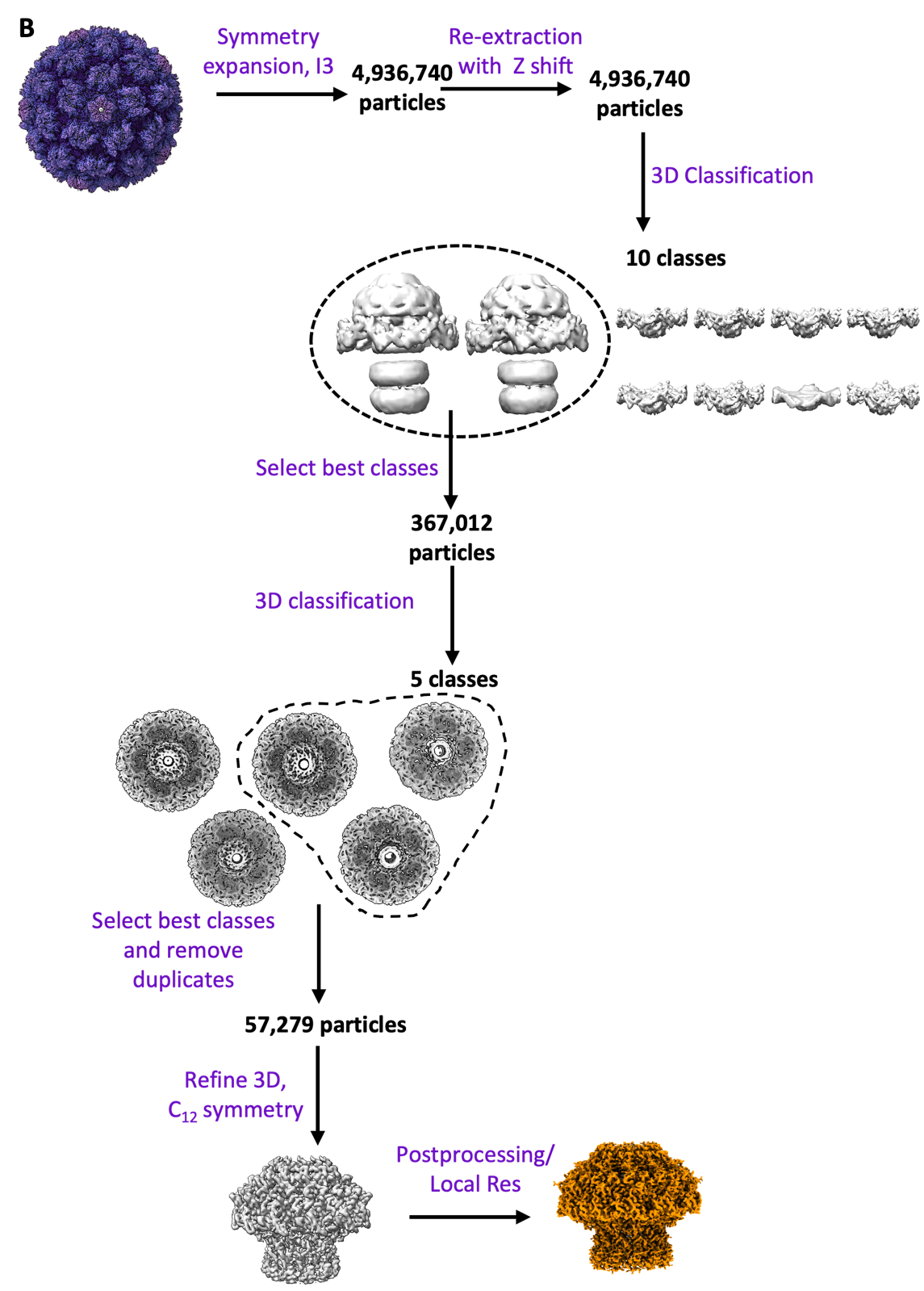
**

**
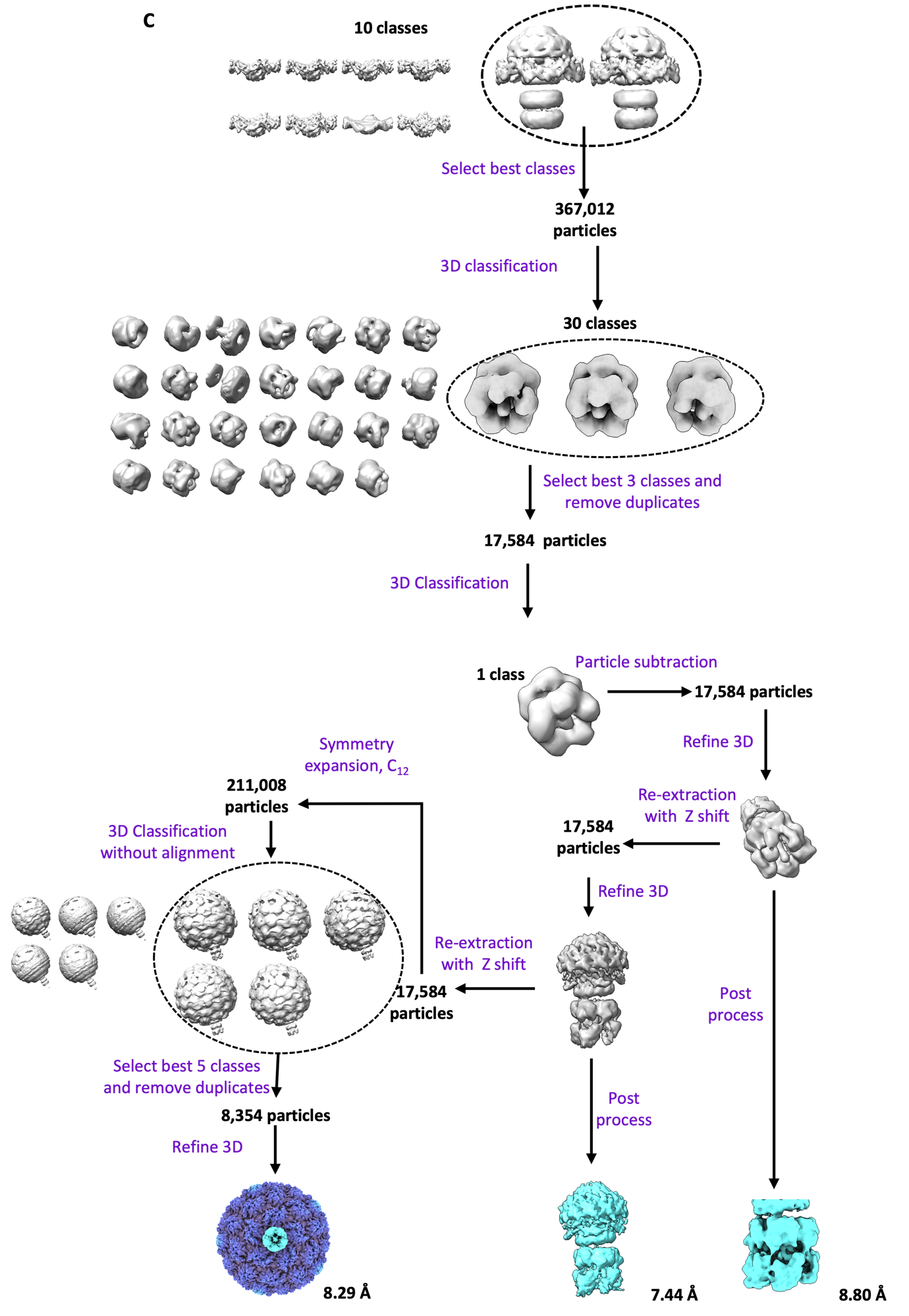
**

***Table S2: Model refinement statistics for the prohead and portal protein***

| **Prohead** | | | | |
| --- | --- | --- | --- | --- |
| Symmetry | I3 | | | |
| Resolution (FSC 0.143) | 3.06 | | | |
| Map-to-model correlation | 0.79 | | | |
| MolProbity score | 1.74 | | | |
| RMS deviations |  |  |  |  |
| Bond lengths / Å | 0.003 | | | |
| Bond angles / ^o^ | 0.6 | | | |
| Ramachandran plot / % |  |  |  |  |
| Favored | 94.1 | | | |
| Allowed | 5.7 | | | |
| Outlier | 0.2 | | | |
| **Portal protein** | | | | |
| Symmetry | C12 | | | |
| Resolution (FSC 0.143) | 2.98 | | | |
| Map-to-model correlation | 0.70 | | | |
| MolProbity score | 1.98 | | | |
| RMS deviations |  |  |  |  |
| Bond lengths / Å | 0.004 | | | |
| Bond angles / ^o^ | 0.7 | | | |
| Ramachandran plot / % |  |  |  |  |
| Favoured | 90.7 | | | |
| Allowed | 9.0 | | | |
| Outlier | 0.3 | | | |
